# Targeting AXL overcomes resistance to docetaxel therapy in advanced prostate cancer

**DOI:** 10.18632/oncotarget.17026

**Published:** 2017-04-11

**Authors:** Jian-Zhong Lin, Zeng-Jun Wang, Wei De, Ming Zheng, Wei-Zhang Xu, Hong-Fei Wu, Alex Armstrong, Jia-Geng Zhu

**Affiliations:** ^1^ Department of Urology, BenQ Medical Center, Nanjing Medical University, Nanjing, China; ^2^ Department of Urology, The First Clinical College of Nanjing Medical University, Nanjing, China; ^3^ Department of Biochemistry and Molecular biology, Nanjing Medical University, Nanjing, China; ^4^ Jiangsu Key Laboratory of Molecular and Translational Cancer Research, Cancer Institute of Jiangsu Province, Nanjing, China; ^5^ Department of Pharmacology, University of Manchester, Manchester, England; ^6^ Department of Urology, Nanjing First Hospital, Nanjing Medical University, Nanjing, China

**Keywords:** AXL, docetaxel resistance, prostate cancer, epithelial-mesenchymal transition (EMT), ATP-binding cassette B1 (ABCB1)

## Abstract

Resistance to docetaxel is a major clinical problem in advanced prostate cancer. The overexpression of AXL receptor tyrosine kinase (AXL) has been correlated with chemotherapeutic drug resistance. However, the role of AXL expression in docetaxel resistance in prostate cancer is yet unclear. In this study, we demonstrate that AXL is overexpressed and activated independent of Gas6 in docetaxel-resistant prostate cancer cells (PC3-DR and DU145-DR). Moreover, we show that forced overexpression of AXL in PC3 and DU145 cells is sufficient to induce resistance to docetaxel in these cell lines. Notably, genetic or pharmacologic inhibition of AXL in the resistant models suppressed cell proliferation, migration, invasion, and tumor growth, and these effects were significantly augmented when AXL inhibition was combined with docetaxel treatment. Mechanistically, we found that AXL inhibition led to reversion of the epithelial-mesenchymal transition (EMT) phenotype and decreased the expression of ATP-binding cassette B1 (ABCB1). Overall, our results identify AXL as an important mediator of docetaxel resistance in prostate cancer. We propose that AXL-targeted therapy, in combination with docetaxel, has the potential to improve the response to docetaxel therapy and reduce resistance induced by prolonged docetaxel therapy in prostate cancer.

## INTRODUCTION

Prostate cancer is the most frequently diagnosed noncutaneous tumor and the second leading cause of cancer-related deaths in men [1, 2]. Although many patients with prostate cancer are initially responsive to anti-androgen therapy, most patients eventually develop advanced castration-resistant prostate cancer (CRPC). While docetaxel is considered a standard first-line therapy in such cases [3], it only gives a modest survival advantage as patients eventually acquire resistance to docetaxel [4]. In addition, docetaxel resistance is often associated with poor prognosis and limited treatment options in advanced prostate cancer [5–6]. Therefore, new treatment modalities are being actively pursued with novel agents, alone or in combination with the existing chemotherapeutic agents, to extend the survival of these patients.

AXL receptor tyrosine kinase (AXL) is a member of the TAM (TYRO3, AXL, MER) family of receptor tyrosine kinases (RTK), activated through several mechanisms, including binding of their ligands, growth arrest-specific 6 (Gas6) [7–9], extracellular domain-mediated dimerization [10], or crosstalk with the extracellular domains on neighboring cells [11]. Studies have indicated that AXL overexpression and activation plays important roles in cell proliferation, migration, and invasion of tumor cells in many malignancies [12]. In addition, elevated AXL expression has been found to be correlated with adverse prognosis and distant metastasis in some cancers [13, 14]. Recently, increased expression or abnormal activation of AXL has particularly been implicated in resistance to cancer chemotherapy and targeted therapy. Importantly, pharmacological inhibition or genetic knockdown of AXL restored sensitivity to these drugs, indicating the role of AXL in drug resistance [15–22]. Collectively, these data indicate that AXL may function as a potent oncogene that can improve resistance to conventional and targeted cancer therapies.

Despite many reports on the function of AXL in drug-resistance, the role of AXL overexpression in the treatment of prostate cancer using docetaxel has been poorly discussed. In this study, we showed that AXL was highly overexpressed and activated in docetaxel resistant PC3 and DU145 cells (PC3-DR and DU145-DR) in a Gas6- independent manner. Furthermore, AXL inhibition augmented the antitumor effect of docetaxel both *in vitro* and *in vivo*. Thus, this work indicates that elevated AXL expression can mediate docetaxel resistance and provide a rationale for the clinical evaluation of anti-AXL therapeutics for the treatment of docetaxel-resistant prostate cancer.

## RESULTS

### AXL is upregulated and activated independent of Gas6 in docetaxel-resistant prostate cancer cells

**Figure 1:**
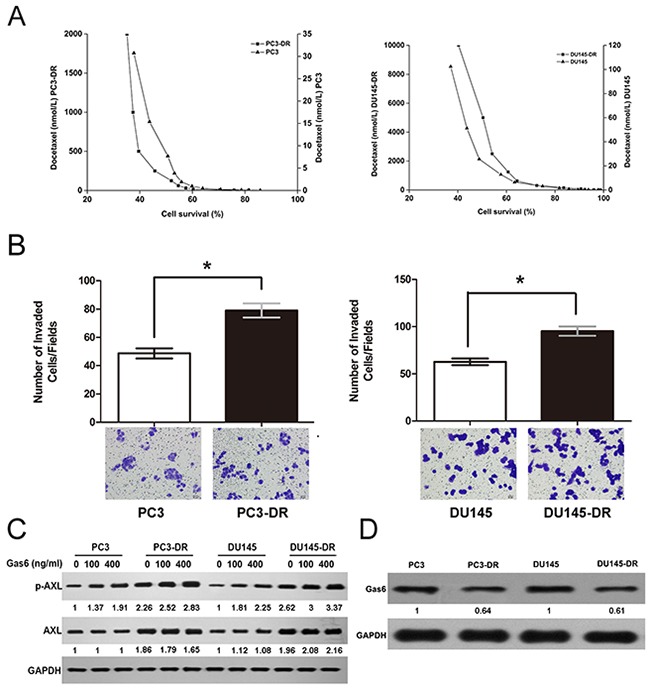
AXL is upregulated and activated independent of Gas6 in docetaxel-resistant cells **(A)** PC3-DR/DU145-DR and the parental cells were treated with the indicated concentrations of docetaxel for 24 h. Cell proliferation was examined using an MTT-based assay. Cell survival is expressed as the percentage of viable docetaxel-treated cells relative to the untreated control cells. **(B)** For cell invasion assay, cells (1 × 10^4^ per well) were seeded in transwells in serum-free medium and the complete growth medium was added to the bottom wells. After 24 h, the invading cells were stained and counted as described. The numbers of resistant and parental cells from four independent experiments were compared. *p < 0.05. **(C)** Cells were treated with increasing doses of Gas6 (100 and 400 ng/ml) for 24 h, and the levels of AXL and p-AXL were analyzed using western blotting. GAPDH was used as the loading control. Gas6 protein levels were normalized to the respective GAPDH levels and then reported below each gel as relative to 0 ng/ml Gas6 in PC3 and PC3-DR cells or DU145 and DU145-DR cells **(D)** Gas6 protein expression in the resistant and parental cells is shown using a representative immunoblot from three independent experiments. GAPDH was used as the loading control. Gas6 protein levels in PC3-DR and DU145-DR, normalized to the respective GAPDH levels, are reported below each lane and then reported below each gel as relative to PC3 and DU145.

To investigate the expression and activation of AXL in docetaxel-resistant prostate cancer, we established two docetaxel-resistant prostate cancer cell lines (PC3-DR and DU145-DR) by culturing PC3 and DU145 cells in docetaxel in a dose-escalation manner. In this study, cell growth assay was conducted to test the effect of docetaxel treatment on the viability of the parental and resistant cells. As shown in Figure 1A, PC3 and DU145 cells were sensitive to docetaxel treatment, with a half-maximal inhibitory concentration (IC50) of 8 and 25 nmol/L, respectively, whereas the resistant cells had significantly higher IC50 values of 168 nmol/L and 5022 nmol/L, indicating ~21-fold and 200-fold higher resistance to docetaxel than the parental cells, respectively. The resistant cells maintained their docetaxel resistance when grown for several weeks in a docetaxel-free media and acquired an irregular shape and a smaller size as compared to the sensitive cells (Supplementary Figure 1). Besides having a distinct morphology, the resistant cells also had increased invasion rates when compared to the parental cells (Figure 1B). Moreover, both the resistant cell lines exhibited higher levels of phosphorylated AXL (phospho-AXL, p-AXL) and total AXL compared to those of the parental cells. To determine whether AXL activation was dependent on Gas6 binding, we treated cells with increasing doses of Gas6. Our results indicated that upon increasing Gas6 dosage, p-AXL levels increased markedly in the parental cells but were only slightly elevated in the corresponding resistant cells (Figure 1C). In addition, Gas6 protein levels in the resistant cells were found to be lower than their levels in the parental cells (Figure 1D). Collectively, the data demonstrate that AXL is clearly upregulated and the constitutive activation of AXL is independent of Gas6 in docetaxel-resistant prostate cancer cells.

### Resistance to docetaxel in prostate cancer cells is associated with AXL levels

**Figure 2:**
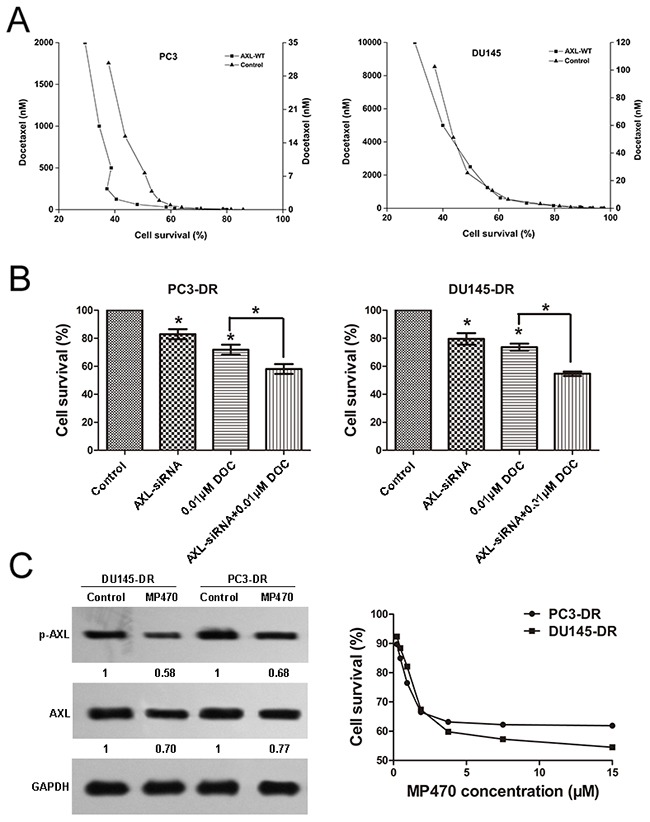
Resistance to docetaxel in prostate cancer cells is associated with AXL **(A)** AXL overexpression renders the PC3 and DU145 cells less sensitive to docetaxel (DOC): PC3 and DU145 cells were transfected with AXL cDNA, using lipofectamine 2000 in 96-well plates. At 72 h after transfection, the cells were confirmed to express higher levels of AXL and treated with DOC. Cell growth assay was performed and the results are expressed as the percentage of viable treated cells relative to the untreated cells. **(B)** AXL knockdown in the PC3-DR and DU145-DR cells sensitizes the cells to DOC: PC3-DR and DU145-DR cells were transiently transfected with siRNA oligonucleotides targeting AXL using lipofectamine 2000. At 72 h after transfection, the cells were confirmed to express lower levels of AXL and treated with DOC. Cell growth assay was performed to examine the effect of the treatment on cell proliferation. *p < 0.05. **(C)** The resistant cells were treated with MP470 (1.875 μM) for 72 h and cell proliferation was evaluated. The expression of AXL and p-AXL in these cells was examined by western blotting. Three independent experiments were performed. GAPDH was used as the loading control. Protein levels, normalized to the respective GAPDH levels, are reported below each gel and then reported below each gel as relative to untreated cells.

Having identified that AXL was overexpressed in the PC3-DR and DU145-DR cells, we further investigated whether genetic upregulation of AXL led to docetaxel resistance in prostate cancer cells. We transiently transfected the PC3 and DU145 cells with the wild-type AXL plasmid for 72 h and then treated the cells with docetaxel for 72 h. The increased AXL expression was confirmed by western blotting (Supplementary Figure 2). This was associated with the emergence of resistance to docetaxel, indicated by increased IC50 values of 54 nmol/L and 2026 nm/L (Figure 2A) in the PC3 and DU145 cells, respectively, suggesting that forced AXL overexpression undermined the growth inhibition effects induced by docetaxel. To further assess the role of AXL in docetaxel resistance, we knocked down AXL using siRNA in DU145-DR cells (Supplementary Figure 3) and found that AXL gene knockdown in these cells sensitized them to docetaxel (Figure 2B). Next, we sought to validate our genetic findings using a commercially available small molecule inhibitor of AXL, amuvatinib (MP470). The treatment of resistant cells with MP470 (1.875 μM) resulted in a marked suppression of AXL expression and cell proliferation (Figure 2C). To explore the synergistic effects of MP470 in combination with docetaxel, we conducted a combination index (CI) analysis in the two resistant cells. We found that pretreatment with MP470 was synergistic with subsequent docetaxel treatment at 50%, 75%, and 90% effective concentrations (EC50, EC75, and EC90, Table 1a), and this was further confirmed by another AXL specific inhibitor, R428 (Table 1b). Taken together, the genetic and pharmacological data indicate that AXL is required for acquirement of docetaxel resistance.

**Table 1A:** Combination Index for Docetaxel and MP470 in DU145-DRand PC3-DR cells

Drug Combination	CI Values at ED50	CI Values at ED75	CI Values at ED90
**DOC and MP470 in DU145-DR(1:6)**	0.545	0.592	0.698
**DOC and MP470 in PC3-DR(1:30)**	0.276	0.348	0.440

(Combination index values (CI) values were calculated using CalcuSyn software CI>1 antagonism, CI=1, additive, CI<1, synergy)

**Table 1B:** Combination Index for Docetaxel and R428 in DU145-DR and PC3-DR cells

Drug Combination	CI Values at ED50	CI Values at ED75	CI Values at ED90
**DOC and R428 in DU145-DR (1:10)**	0.337	0.414	0.542
**DOC and R428 in PC3-DR (1:100)**	0.213	0.383	0.436

(Combination index values (CI) values were calculated using CalcuSyn software CI>1 antagonism, CI=1, additive, CI<1, synergy)

### AXL inhibition effectively induces apoptosis and reduces the migration and invasion of docetaxel-resistant prostate cancer cells

**Figure 3:**
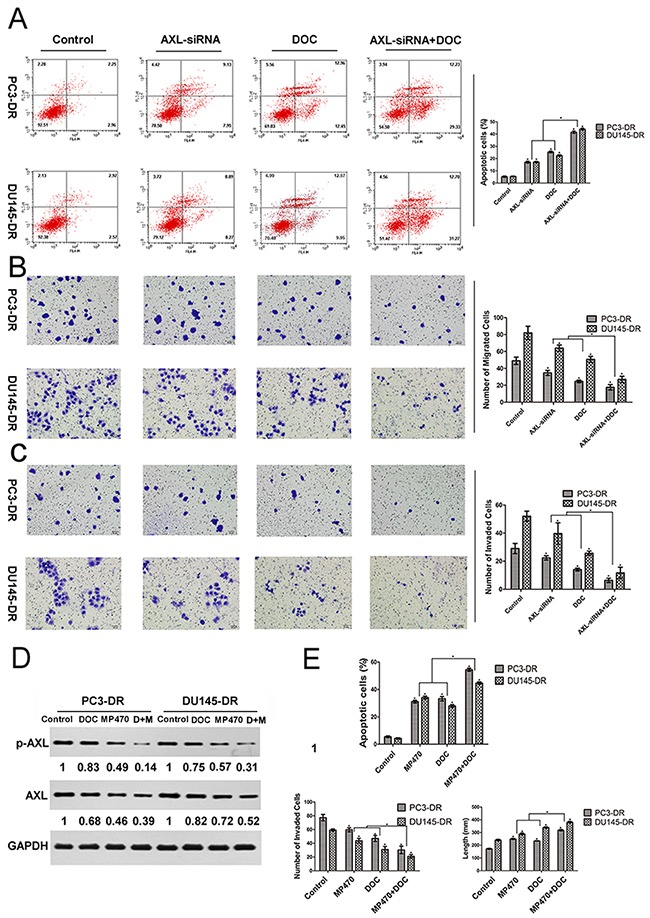
AXL inhibition effectively induces apoptosis and reduces the migration and invasion of docetaxel-resistant prostate cancer cells PC3-DR and DU145-DR cells were left untreated (control) or treated with AXL-siRNA, docetaxel (DOC, 0.01 μM for PC3-DR and 0.1 μM for DU145-DR), or a combination of both for 24 h. **(A)** The apoptotic cell death was analyzed by fluorescence-activated cell sorting (FACS). **(B)** and **(C)** Transwell migration and invasion assays were performed to compare and quantify the migratory and invasive capabilities of the resistant cells. Cells were seeded into transwell chambers and those that passed through the Matrigel-coated polycarbonate membrane were fixed, stained with eosin staining solution, and examined using light microscopy at × 200 magnification. **(D)** and **(E)** The resistant cells were treated with MP470 (1.875 μM), DOC, or a combination of both (DOC+MP470, D+M). The levels of AXL and p-AXL were then analyzed using western blotting. Protein expression was quantified using the Gel-Pro 32 software. GAPDH was used as the loading control. Protein levels, normalized to the respective GAPDH levels, are reported below each gel and then reported below each gel as relative to untreated cells. Simultaneously, FACS, wound-healing, and transwell assays were employed to quantify the apoptosis, migration, and invasion of the resistant cells, respectively. Three independent experiments were performed and quantitation was performed using the Image-Pro Plus 6.0 software. All data points are represented as mean ± SEM, *p < 0.05 indicates a significant difference.

Based on the above findings, we further examined whether AXL inhibition was also effective in inducing apoptosis and suppressing the migration and invasion of cells. Fluorescence-activated cell sorting (FACS) analyses indicated that both siRNA-mediated inhibition of AXL and docetaxel treatment were effective in inducing apoptosis in PC3-DR and DU145-DR cells (Figure 3A). Further, a combination of AXL inhibition with docetaxel treatment induced a higher apoptotic effect on these cells than the single treatments. We further explored whether AXL upregulated the migratory and invasive potential of the resistant cells by transwell assays. Our studies demonstrated that AXL knockdown significantly inhibited the migratory and invasive capacity of the resistant cells as compared with control cells. A greater suppression was observed when AXL inhibition was combined with docetaxel treatment (Figure 3B and 3C). In addition, we sought to validate the above genetic findings using an AXL inhibitor. Based on the concentration-response growth curves and apoptosis analysis, the doses of docetaxel (0.01 μM for PC3-DR and 0.1 μM for DU145-DR) and MP470 (1.875 μM for both cells) that produced ~30% growth inhibition were chosen for use in the combination experiments (Figure 1A, Figure 2C and Supplementary Figure 4). As shown in Figure 3D, treatment with either drug significantly reduced the AXL and p-AXL protein levels. The combination treatment with both the drugs exhibited a stronger suppression of AXL and p-AXL expression as compared to the single treatments (Figure 3D). As expected, the effects of the pharmacologic inhibition of AXL on both the resistant cells further validate the genetic-inhibition results (Figure 3E). Overall, these investigations indicate that the resistant cells were reliant on AXL for apoptosis, cell migration, and invasion.

### AXL inhibition restores docetaxel sensitivity in DU145-DR xenograft tumors

**Figure 4:**
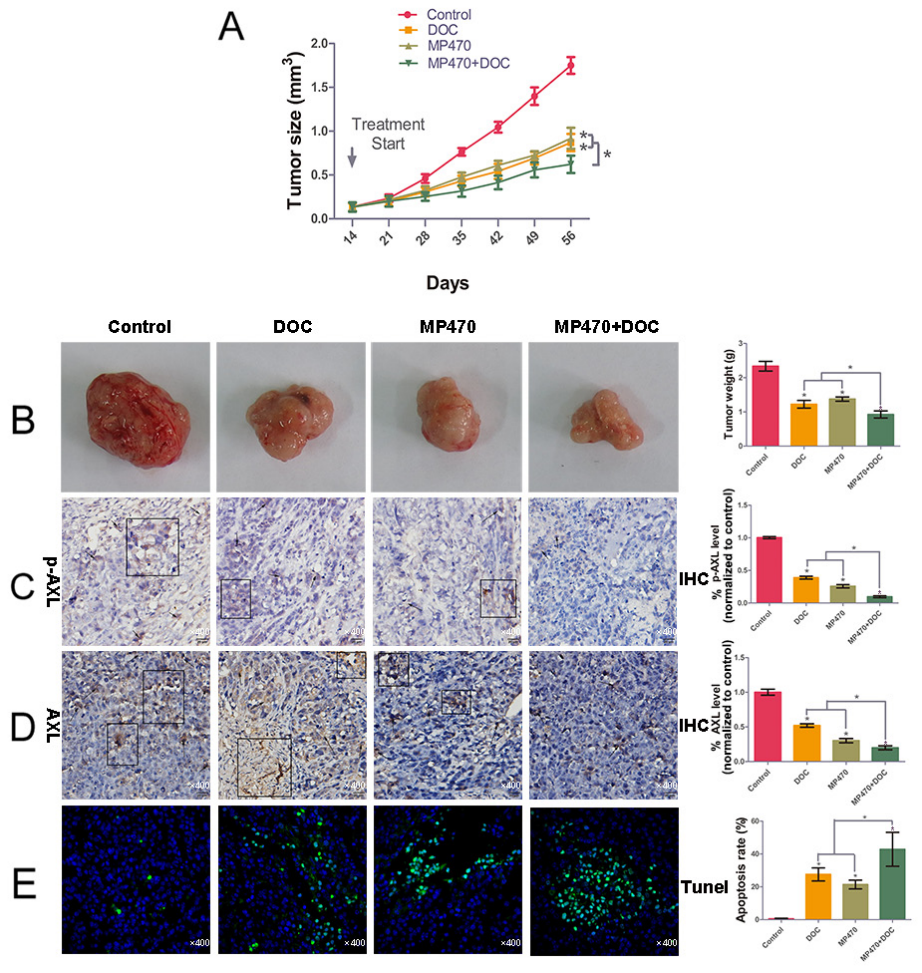
AXL inhibition restores docetaxel sensitivity in DU145-DR xenograft tumors We performed the *in vivo* determination of the growth-inhibitory effects of MP470 and docetaxel (DOC) on DU145-DR cell xenografts. DU145-DR cells (5 × 10^6^ cells/50 μl culture medium were mixed with an equal amount of BD Matrigel™ in a final volume of 100 μl) were subcutaneously implanted in athymic nude mice. When the tumor volumes reached a similar size of ~0.1-0.15 cm^3^, the mice were treated with DMSO (control), MP470 (60 mg^-1^·kg^-1^·d for 14 days by oral gavage), DOC (10 mg/kg, given intraperitoneally two days per week, for two consecutive weeks), or a combination of MP470 with DOC. **(A)** and **(B)** Tumor volume, plotted as a function of time, and tumor weight for the various treatments are shown. Pictures of DU145-DR xenograft tumors after six weeks of implantation are shown for mice left untreated (control) or treated with MP470, DOC, or a combination of both. **(C)** and **(D)** Immunohistochemical analyses of AXL and p-AXL. Arrow indicates the positive immunostaining expression (×400). Pathologic IHC quantitation was determined via IOD values. **(E)** Images showing DNA fragmentation (green staining) by the terminal deoxynucleotidyl transferase dUTP nick end labeling (TUNEL) assay and counterstaining with DAPI (green/blue; hybrid cyan) in tumor sections. Photomicrographs are shown at a similar magnification of 400×. Left: Representative photomicrographs. Right: Quantitative analysis. Data are expressed as mean ± SEM. *p < 0.05 indicates a significant difference.

To further explore the effect of AXL inhibition on docetaxel resistance *in vivo*, a xenograft study was performed to determine the therapeutic effects of MP470 and docetaxel, alone or in combination. Our results indicated that 60 mg/kg MP470 or 10 mg/kg docetaxel effectively inhibited tumor growth and lowered the weight of DU145-DR xenografts as compared to the untreated xenografts in athymic nude mice (Figure 4A and 4B). The immunohistochemistry (IHC) analyses of DU145-DR tumor specimens indicated that MP470 or docetaxel alone significantly inhibited the expression of AXL in DU145-DR xenografts (Figure 4C and 4D). Moreover, the combined treatment of MP470 and docetaxel led to lower AXL expression, particularly that of p-AXL, which was in agreement with the *in vitro* results (Figure 4C and 4D). Further, the combination treatment was more effective than the single drug treatments in suppressing tumor growth (Figure 4A and 4B) and inducing tumor apoptosis in xenografts as detected by the terminal deoxynucleotidyl transferase dUTP nick end labeling (TUNEL) assay (Figure 4E). Our results thus demonstrate that AXL inhibition restored docetaxel sensitivity *in vivo*.

### AXL-mediated docetaxel resistance is associated with EMT phenotypes

**Figure 5:**
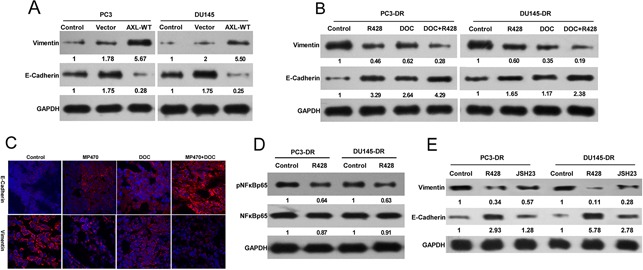
AXL-mediated docetaxel resistance is related to EMT phenotypes **(A)** PC3-DR and DU145-DR cells were treated with R428 (1 μM) for 24 h. Cell lysates were examined by western blotting with specific antibodies as indicated. **(B)** PC3 and DU145 cells were transfected with AXL cDNA using lipofectamine 2000 in 96-well plates. Twenty-four hours after transfection, the levels of E-cadherin and vimentin were analyzed by western blotting and compared with that in the control cells. **(C)** The resistant cells were treated with R428, docetaxel (DOC), or a combination of both. E-cadherin and vimentin protein expression was evaluated by western blotting. **(D)** The expression of E-cadherin and vimentin in DU145-DR xenograft tumors treated with MP470, DOC, or their combination was examined by immunofluorescence (400×). **(E)** The expression of E-cadherin and vimentin in the resistant cells after treatment with 25 μM JSH-23 (an NF-κB inhibitor) was compared with that in untreated cells using western blotting. Three independent experiments were performed. GAPDH was used as the loading control. Protein levels normalized to the respective GAPDH levels and as relative to untreated cells are reported below each gel.

We further sought to identify the signaling events downstream of AXL that might promote the acquired resistance to docetaxel in prostate cancer. R428, a specific AXL inhibitor, which markedly suppressed AXL expression and induced apoptosis in the resistant cell lines (Supplementary Figure 5), was used to examine the impact of AXL inhibition on the signaling pathways. We found that R428 treatment clearly reduced the phosphorylation levels of the extracellular signal-regulated protein kinases 1 and 2 (ERK1/2), and protein kinase B (PKB, also known as AKT) in the resistant cells, whereas their total expression levels remained unchanged (Supplementary Figure 6). To test whether AXL played a role in EMT induction, we first assessed the effects of AXL overexpression on the expression of EMT marker proteins. Forced overexpression of AXL in PC3 and DU145 cells significantly enhanced the levels of the mesenchymal marker, vimentin, and reduced the expression of the epithelial marker, epithelial cadherin (E-cadherin, Figure 5A). In addition, the inhibition of AXL using R428 was found to upregulate E-cadherin and downregulate vimentin in both the resistant cells. The combination treatment of R428 with docetaxel induced higher E-cadherin and lower vimentin expression as compared to the single drug treatments (Figure 5B). The effect of AXL inhibition by MP470 *in vivo* further confirmed the *in vitro* findings (Figure 5C). To further elucidate the mechanism of AXL regulation of EMT induction, we focused on the nuclear factor kappa-B (NF-κB) pathway, since this pathway has been proven to be a downstream target of AXL activation [7, 15]. The resistant cells were treated with R428 and the NF-κB activation inhibitor II (JSH-23), and their effects on the expression of EMT markers were analyzed. Our results showed that AXL inhibition by R428 markedly decreased the phosphorylation level of NF-κB p65, and NF-κB inhibition by JSH-23 led to an increase in E-cadherin and a decrease in vimentin levels (Figure 5D). Taken together, the data suggest that AXL upregulation activates AKT, ERK, or NF-κB signaling to promote resistance to docetaxel treatment in prostate cancer, perhaps in association with the acquisition of EMT. The NF-κB pathway may also be involved in AXL-induced EMT phenotype in docetaxel-resistant prostate cancer.

### AXL-mediated resistance occurs with ABCB1 upregulation

**Figure 6:**
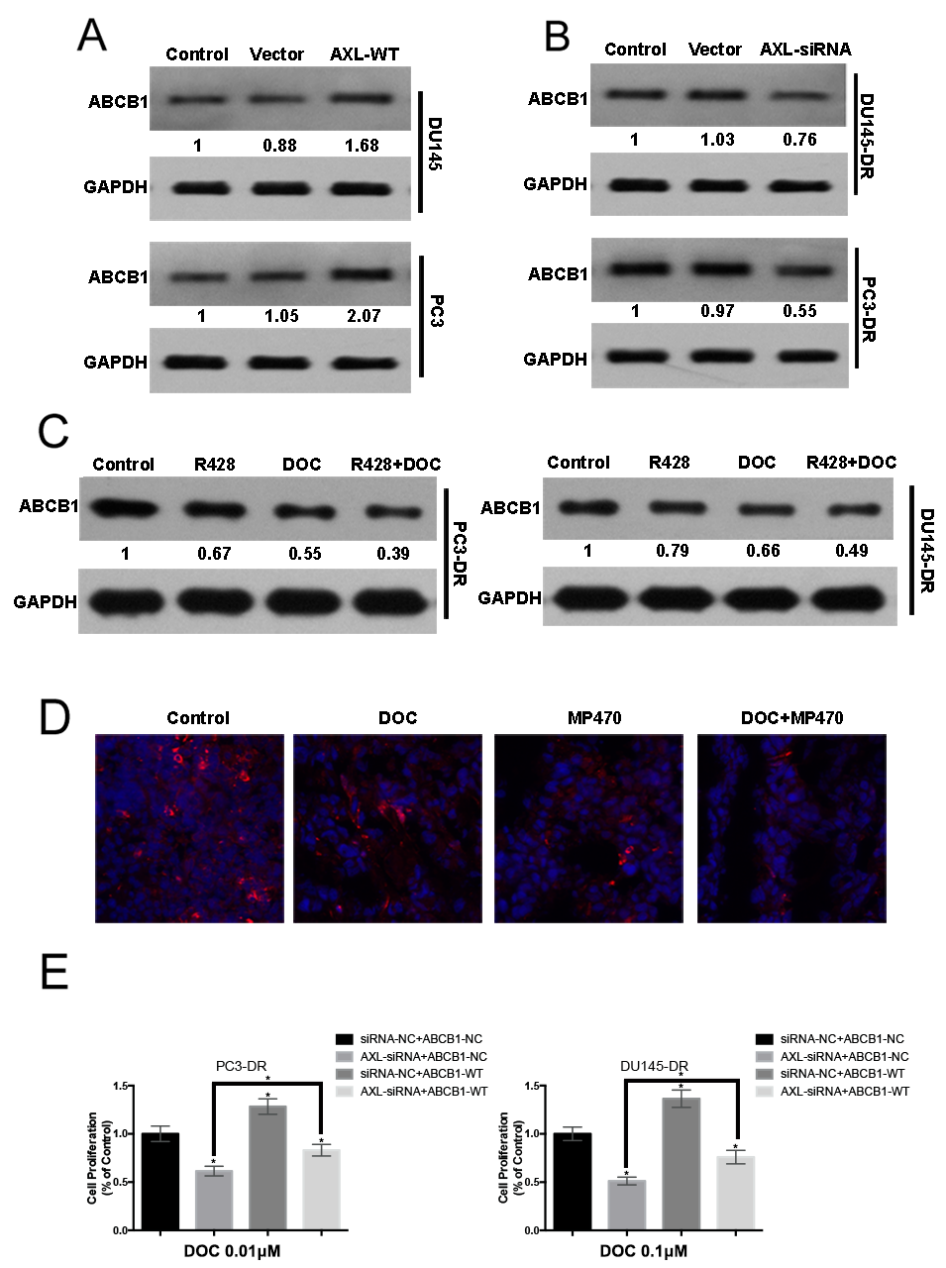
AXL-mediated resistance occurs with ABCB1 upregulation **(A)** PC3 and DU145 cells were transfected with AXL cDNA, using Lipofectamine 2000 in 96-well plates. Twenty-four hours after transfection, ABCB1 expression was examined by western blotting and compared with that in the control cells. **(B)** PC3-DR and DU145-DR cells were transiently transfected with AXL-siRNA and ABCB1 expression was examined by western blotting. **(C)** The expression of ABCB1 in the resistant cells treated with R428, docetaxel (DOC), or a combination of both was analyzed by western blotting. **(D)** Immunofluorescence analyses for ABCB1 were performed after the treatment of mice with MP470, DOC or a combination of both. **(E)** PC3-DR and DU145-DR cells were co-transfected with siRNA-NC/siRNA-AXL and Vector-NC/ABCB1-WT and then treated with the indicated dose of docetaxel for 48h. Cell proliferation was evaluated using MTT assay. Three independent experiments were performed. GAPDH was used as the loading control. Protein levels, normalized to the respective GAPDH levels and as relative to untreated cells are reported below each gel.

Overexpression of ABCB1 is regarded as an important mechanism involved in the acquisition of docetaxel resistance in prostate cancer. In our study, exogenous AXL overexpression in the PC3 and DU145 cells was shown to induce a higher ABCB1 expression than in the parental cells (Figure 6A). In addition, AXL inhibition by siRNA led to a marked decrease in the ABCB1 levels in the resistant cell lines (Figure 6B). Moreover, a similar lowering of ABCB1 expression was observed *in vitro* upon treatment with R428. Interestingly, the combined treatment of R428 with docetaxel induced further lowering of ABCB1 expression compared to treatment with either drug alone (Figure 6C). Additionally, *in vivo* immunofluorescence microscopy indicated that AXL inhibition significantly lowered the ABCB1 levels, further corroborating our *in vitro* observations (Figure 6D). We next determine whether ABCB1 was functionally involved in AXL-mediated docetaxel resistance, The results indicated that ABCB1 overexpression partly recapitulated the docetaxel resistance in AXL-knockdown-resistant cells (Figure 6E). Collectively, our findings suggest that ABCB1 upregulation may be another mechanism of AXL-mediated docetaxel resistance in prostate cancer.

## DISCUSSION

Docetaxel therapy has yielded clinical benefits for advanced prostate cancer; however, both intrinsic and acquired resistance are common outcomes. Multiple mechanisms of docetaxel resistance exist in prostate cancer, including ABC transporters [23, 24], glucocorticoid receptor (GR) [25], androgen receptor (AR) splicing [26, 27], epithelial plasticity [28, 29], and stem cells [30]. A better understanding of the mechanisms by which docetaxel resistance develops in prostate cancer can enable the development of improved treatment strategies. Recent studies have found high levels of AXL expression in advanced human prostate cancer tissue [8]. Furthermore, *in vitro* studies suggest that AXL signaling is associated with prostate cancer development and progression [8, 31]. The study herein is the first to describe a role of AXL in resistance to docetaxel both *in vitro* and *in vivo*, and thus, provides a rationale for the development and use of anti-AXL therapeutics for the treatment of docetaxel-resistant prostate cancer.

Docetaxel resistance is challenging to study owing to the lack of access to patient tissue upon relapse. To model docetaxel mechanisms that may occur in humans, several models of acquired resistance have been established via prolonged exposure of sensitive cells to docetaxel [29, 30, 32]. Some models indicated that resistant clones and tumors had an increased expression of and dependency on AXL [15–22]. In the current study, AXL was found to be overexpressed and activated in the PC3-DR and DU145-DR cells as compared to the parental cells. Activation of AXL often occurs upon binding to Gas6, including in prostate cancer [7, 8, 12]. However, in our study, although elevated AXL activation was found in the resistant cells, their Gas6-responsiveness was not correspondingly noticeably improved. Furthermore, lower Gas6 expression was observed in the resistant cells compared with the parental cells, suggesting a possibility of Gas6-independent AXL activation. The data may be explained by prior studies in which overexpression of AXL and treatment with drugs caused ligand-independent autoactivation of AXL [10, 33]. Moreover, our studies also demonstrated that forced overexpression of AXL in the PC3 and DU145 cells markedly improved their capacity to resist the toxic effects of docetaxel, indicated by increased IC50 values. Taken together, these results indicate that AXL overexpression and activation, independent of Gas6, is closely related to docetaxel resistance in prostate cancer.

Being of therapeutic interest, we investigated whether AXL inhibition is sufficient to reverse the resistance to docetaxel in prostate cancer. Our results demonstrate that AXL inhibition by siRNA partly suppressed the growth and invasiveness, and induced apoptosis in the resistant cells, and the combination of AXL knockdown with docetaxel treatment induced a greater inhibitory effect on the resistant cells. With increasing evidence supporting the role of AXL in resistance to cancer chemotherapy and targeted therapy, many AXL inhibitors, including small molecule inhibitors, anti-AXL monoclonal antibodies, and nucleotide aptamers, are under development [22]. In this study, two AXL inhibitors were tested: MP470, a structure-based multitargeted RTK inhibitor that targets mutant AXL, c-Kit, and the platelet-derived growth factor receptor α (PDGFRα); and R428, a specific small molecule AXL tyrosine kinase inhibitor [22]. In previous studies, both MP470 [15, 34, 35] and R428 [34, 36, 37] were shown to suppress AXL expression and recover drug sensitivity in many models of acquired resistance. MP470 was recently reported for discontinuous phase II clinical development by Astex Pharmaceuticals and R428 is undergoing phase I/II clinical trials by Rigel Pharmaceuticals [38]. Consistent with prior studies, the two inhibitors were demonstrated to significantly reduce the expression of AXL and p-AXL in our study. Moreover, we also demonstrated that the suppression of AXL using inhibitors was sufficient to reverse the resistance to docetaxel in the resistant cells, which further validated the genetic-inhibition effect. Importantly, our *in vivo* results further confirmed our *in vitro* observations. Collectively, our findings suggest that targeting AXL is effective in overcoming docetaxel resistance in prostate cancer.

EMT is typically characterized by the loss of expression of epithelial markers (i.e., E-cadherin) and gain of expression of mesenchymal markers (i.e., vimentin). Several studies have shown that EMT mediates docetaxel resistance in prostate cancer [28, 29]. In this study, we found that AXL-overexpressing PC3-DR and DU145-DR cells exhibited increased migration and adhesion, properties associated with EMT, which were suppressed by AXL inhibition via siRNA and RTK inhibitors. Furthermore, AXL inhibition also led to higher E-cadherin and lower vimentin expression levels both *in vitro* and *in vivo*. These findings were further verified by our observation of forced expression of AXL in PC3 and DU145 cells leading to lower E-cadherin and higher vimentin expression levels. These studies indicate that EMT could be driven by both intrinsic and acquired increase in AXL. Moreover, our data also suggest that activation of multiple pathways including AKT, ERK, and NF-kB may promote docetaxel resistance downstream of AXL upregulation in the resistant cells. This is in concordance with prior studies indicating that AXL can drive the growth of cancer cells through the activation of each of these pathways [15, 16, 34, 39, 40]. Additionally, our studies further show that NF-kB maybe involved in AXL-mediated EMT regulation. Therefore, our findings suggest that induction of EMT contributes to the AXL-mediated acquired docetaxel resistance in prostate cancer. Further work will be necessary to completely elucidate the mechanisms by which AXL might promote resistance in prostate cancer through the induction of EMT.

ABCB1, a verified mechanism underlying docetaxel resistance in prostate cancer, is induced by docetaxel and diminishes its efficacy by transporting it across the cell membrane [23, 24]. Our results indicated a marked upregulation of ABCB1 in resistant cells, which is consistent with prior studies (data not shown). Our study also showed that forced expression of AXL in the PC3 and DU145 cells led to an increased ABCB1 level, suggesting the possible association of ABCB1 with AXL overexpression. Some RTK inhibitors have been shown to inhibit the drug efflux function of ABCB1, thus overcoming the resistance of cancer cells to traditional chemotherapeutic drugs [41, 42]. In the current study, our data revealed that the AXL-specific inhibitor, R428, was sufficient to suppress ABCB1 protein expression in the resistant cells. In subsequent xenograft tumors, another AXL inhibitor, MP470, was also found to significantly block ABCB1 expression. Furthermore, downregulation of AXL using R428 or MP470 in combination with docetaxel led to stronger suppression of ABCB1 expression *in vitro* and *in vivo*. Moreover, we also provided evidence that ABCB1 overexpression suppressed docetaxel response in AXL-knockdown-resistant cells. These findings suggest that ABCB1 may be involved in AXL-mediated resistance to docetaxel and thus may be another mechanism underlying docetaxel resistance in prostate cancer. Some studies also indicated that the increase in ABCB1 may be associated with EMT [43, 44]. Taken together, more studies may be necessary to determine the exact roles ABCB1 plays in AXL-mediated resistance to docetaxel in prostate cancer.

Our study has several limitations. We used subcutaneous, not orthotopic, injection to model soft tissue growth. Although orthotopic injection is ideal, it is challenging to model because of the anatomy of the mouse prostate [45]. Another limitation of our study is that we used androgen receptor-negative cell lines, which are commonly used in prostate cancer studies. However, it has been demonstrated that some of the antitumor actions of docetaxel can be attributed to its effect on the androgen receptor axis [27, 28]. Thus, our study did not totally recapitulate the clinical disease, and we propose to use the androgen receptor-positive cell lines, such as VCaP and C4-2B, in future studies.

In summary, our research showed that the inhibition of AXL has an antitumor effect in models of docetaxel-resistant prostate cancer. Furthermore, AXL inhibition had synergistic activity with docetaxel and markedly enhanced docetaxel-mediated cytotoxicity in docetaxel-resistant cells. These findings suggest that AXL inhibitors should be evaluated in clinical trials as adjuvants with docetaxel in the treatment of docetaxel-resistant prostate cancer.

## MATERIALS AND METHODS

### Antibodies and reagents

Antibodies against AXL, p-AXL (Tyr702), Gas6, NF-κB, phospho-NF-κB (p-NF-κB), E-cadherin, vimentin, and ABCB1 were purchased from Santa Cruz Biotechnology (Santa Cruz, CA, USA). Antibodies against AKT, phospho-AKT (p-AKT, ser473), ERK, and phospho-ERK (p-ERK, Thr202/Tyr204) were purchased from Cell Signaling Technology (Danvers, MA, USA). Recombinant Gas6 was purchased from R&D Systems (Minneapolis, MN, USA). JSH-23, MP470, and R428 were purchased from MedChemExpress (Monmouth Junction, NJ, USA).

### Cell culture and establishment of docetaxel-resistant subclones

The human prostate cancer cell lines PC3 and DU145 were purchased from the Cell Bank of Shanghai Life Science Institution, Chinese Academy of Sciences and grown in F12 and RPMI 1640 media, respectively, supplemented with 10% fetal bovine serum (Gibco, Carlsbad, CA, USA) in a humidified incubator (Thermo Scientific, Waltham, MA, USA) at 37°C with 5% CO_2_. The cells were incubated with gradually increasing concentrations of docetaxel, starting at 0.1 nM and 2.5 nM for PC3 and DU145, respectively, and maintained until the docetaxel-sensitive clones died. The surviving cells repopulated and after 10 months, the cells dividing freely in 10 nM and 100 nM docetaxel-containing media were considered as resistant cell lines and labeled as “PC3-DR” and “DU145-DR,” respectively.

### Cell growth and apoptosis assays

Proliferation assays were performed using Cell Proliferation Kit I, MTT (Roche, Indianapolis, IN, USA) according to the manufacturer's protocol. Apoptosis was assessed using the Annexin V-FITC Apoptosis Detection Kit (BioVision, Mountain View, CA, USA) and propidium iodide (PI). Analysis was performed using a FACSCalibur flow cytometer (Becton Dickinson, San Jose, CA, USA).

### Western blot analysis

Cells were serum-starved overnight and whole cell lysates were prepared using the radioimmunoprecipitation assay (RIPA) buffer supplemented with protease inhibitor cocktail. Forty micrograms of total protein was resolved by SDS-PAGE and then transferred to PVDF membranes. The transferred proteins were immunoblotted with primary polyclonal antibodies. Glyceraldehyde-3-phosphate dehydrogenase (GAPDH) was used as the loading control. The protein expression was quantified by densitometry, using the Gel-Pro 32 software.

### Transfection and siRNA technology

PC3 and DU145 cells were transiently transfected with pcDNA-AXL or pcDNA-ABCB1 plasmids, using lipofectamine 2000 (Invitrogen, Carlsbad, CA, USA). Forty-eight hours after transfection, the cells were harvested, and western blotting was performed to confirm the overexpression of AXL or ABCB1. For AXL knockdown, lentiviral-based AXL shRNA constructs were obtained from Open Biosystems (Huntsville, AL, USA). PC3-DR and DU145-DR cells were transiently transfected with shRNA against AXL or the vector control, shGFP, using lipofectamine 2000 (Invitrogen, Carlsbad, CA, USA). Forty-eight hours after transfection, the transfected cells were subject to selection using 1.0 μg/ml puromycin. The transfection efficiency of the cells was monitored by fluorescence microscopy (OLYMPUS IX51×200). Quantitative real time RT-PCR was performed to confirm AXL knockdown.

### Wound-healing assay

Cells were seeded in 6-well culture plates. After the cells reached confluence, the cell layer was scratched with a sterile P-200 pipette tip. The cells were then cultivated in complete medium containing the indicated drugs for 24 h before wound exposure.

### Cell migration and invasion

Cell migration and invasion assays were performed as previously described (8) using a modified transwell chamber migration assay and invasion assay matrigel-coated membrane (BD Biosciences Bedford, MA). Briefly, Approximately 1 × 10^4^ cells suspended in serum-free medium were plated in 24-well plates and treated with the indicated drugs or AXL-siRNA. Twenty-four hours after treatment, cell migration and invasion were measured as per manufacturer's instructions.

### *In vivo* study

All animal use and care protocols followed the guidelines of the Institutional Animal Care and Use Committee and were approved by the Hospital of Nanjing BenQ Medical Center Animal Care and Use Committee. To generate docetaxel-resistant tumors, we injected 5 × 10^6^ DU145-DR cells into the flanks of 4-week-old male athymic nude mice. After the tumors became palpable (volume reached 100-150 mm^3^), 24 (six per group) tumor-bearing mice were randomly picked and treated with the following: MP470 (60 mg^-1^·kg^-1^·d for 14 days by oral gavage), docetaxel (10 mg/kg given intraperitoneally (i.p.) two days per week, for two consecutive weeks), or a combination of both. The tumors resumed growth and six randomly picked mice were treated with saline as control. Tumor tissue was collected for analysis following standard procedures. The tumor sizes and weight were monitored for 6 weeks. Animal health was assessed daily to minimize pain and distress.

### Immunohistochemical (IHC) analyses

The expression levels of p-AXL and AXL were analyzed by IHC as described previously [17]. The images were analyzed using the Leica QWin (Leica Microsystems, Wetzlar, Germany) and Image-Pro Plus (IPP) 6.0 software (Media Cybernetics Corp, Bethesda, MD, USA). Six images per tumor were analyzed via the integrated optical density (IOD) values. Untreated was used as the loading control. Representative fields were photographed under 400× magnification.

### Confocal microscopy analyses

Briefly, following the respective treatments, the tissues were fixed with 4.0% paraformaldehyde for 20 min, washed 3 times with PBS for 10 min each, blocked in 3% H_2_O_2_-methanol for 10 min, and washed again with PBS. Then, the sections were immunostained using antibodies diluted in the blocking buffer, followed by the fluorophore-conjugated secondary antibodies, and washed again with PBS. The fluorescent DNA dye DAPI (4’,6-diamidino-2-phenylindole) was added before the penultimate washing to stain the nuclei. In addition, for the TUNEL assay, the sections were fixed with 4.0% paraformaldehyde, permeabilized with 0.1% Triton X-100 in PBS for 15 min, and blocked in 3% H_2_O_2_-methanol for 15 min. The sections were then washed with PBS, treated with proteinase K for 30 min at 37°C, and then incubated with Streptavidin-FITC in the dark. The nuclei were counterstained using DAPI. Immunofluorescence staining was observed under a confocal laser scanning microscope (LSM 510, Zeiss, Gottingen, Germany) and photographed at a magnification of 400×

### Statistical analysis

Data represent mean ± SEM. Statistical analyses were performed by using the Student's t-test to compare the results. Differences were considered statistically significant if *p<0.05.

## SUPPLEMENTARY MATERIALS FIGURES AND TABLES


