# Supplementary material for: Targeting AXL overcomes resistance to docetaxel therapy in advanced prostate cancer
Source: Oncotarget. 2017 Apr 11;8(25):41064–77. doi: 10.18632/oncotarget.17026 (PMC5522277; doi:10.18632/oncotarget.17026)
Supplement: Supplementary file 1 [file oncotarget-08-41064-s001.pdf]

Targeting AXL overcomes resistance to docetaxel therapy in advanced prostate cancer

SUPPLEMENTARY FIGURES

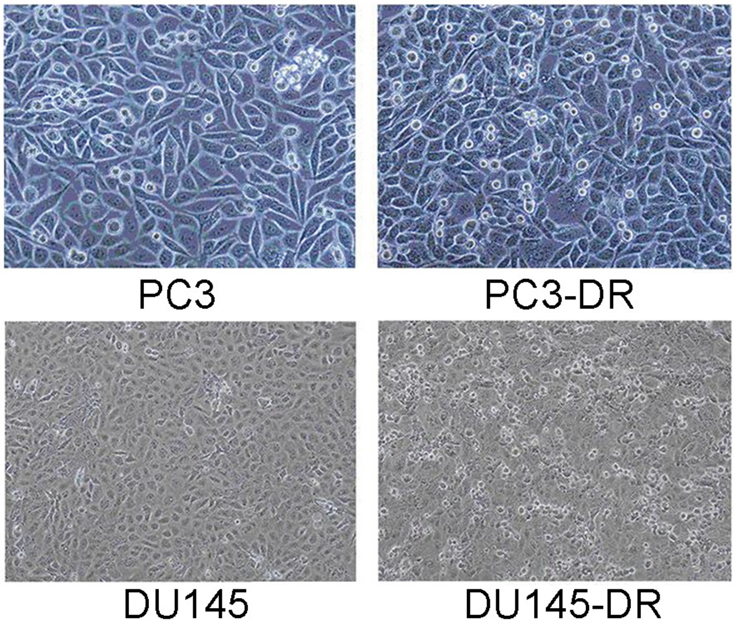

Supplementary Figure 1: The resistant cells acquired an irregular shape and a smaller size as compared to the sensitive cells.

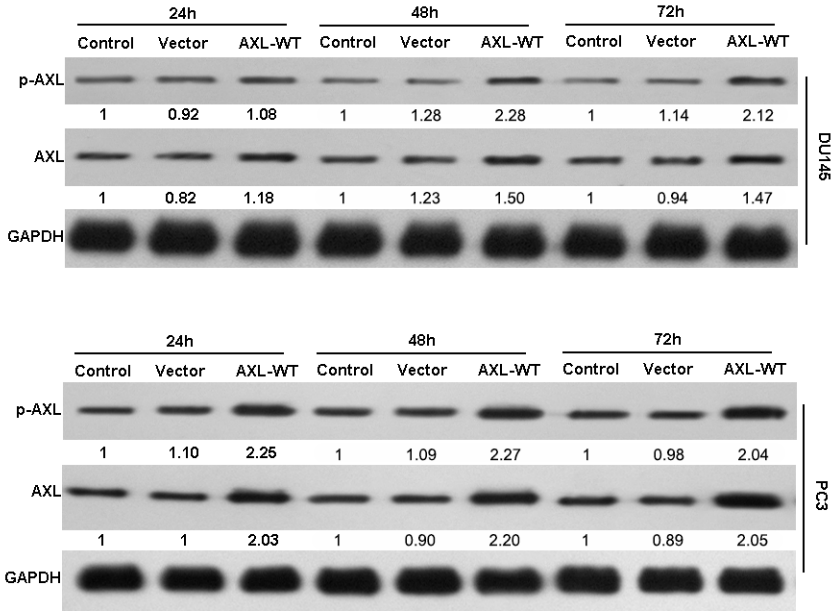

Supplementary Figure 2: PC3 and DU145 cells was transfected with the wild-type AXL plasmid for 24h, 48h, 72h. The increased AXL expression was confirmed by western blotting.

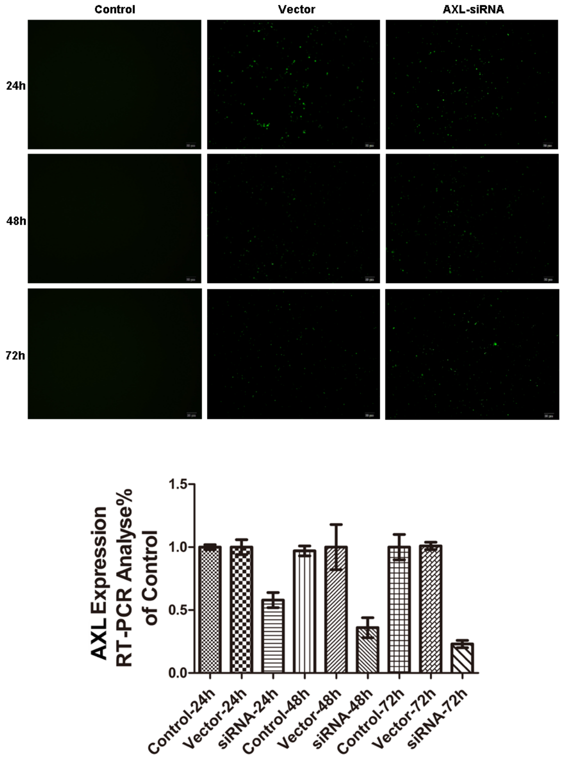

Supplementary Figure 3: AXL was knocked down using siRNA in DU145-DR cells. The transfection efficiency of the cells was monitored by fluorescence microscopy. Quantitative real time RT-PCR was performed to confirm AXL knockdown.

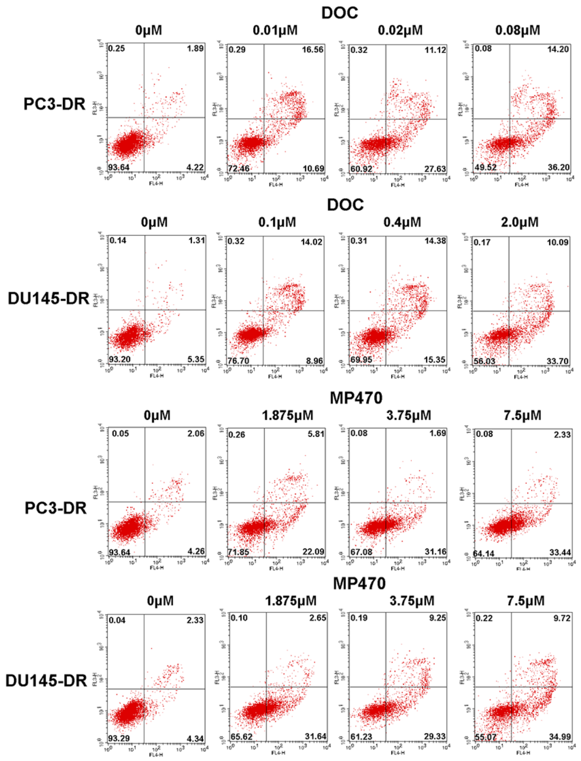

Supplementary Figure 4: PC3-DR and DU145-DR cells were treated with escalating dose levels of MP470 and docetaxle(DOC) for 24h. FACS was used to assess the resistant cells apoptosis after the treatment of MP470 or DOC.

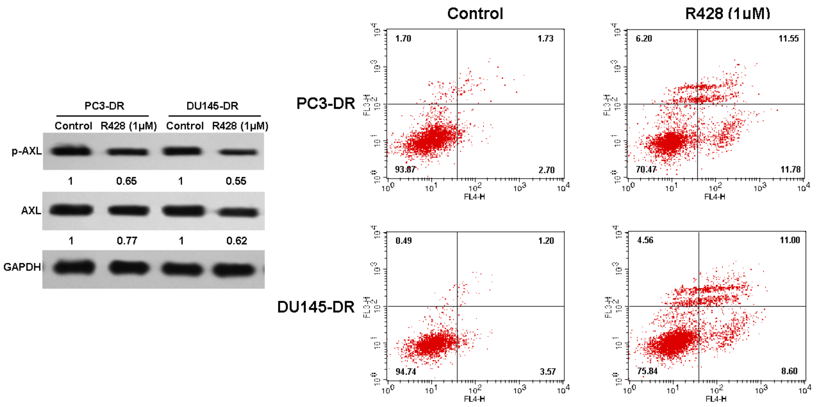

Supplementary Figure 5: The expression of AXL in the PC3-DR and DU145-DR cells treated with R428(1μM) was analyzed by western blotting. FACS was used to assess the resistant cells apoptosis after R428(1μM) treatment.

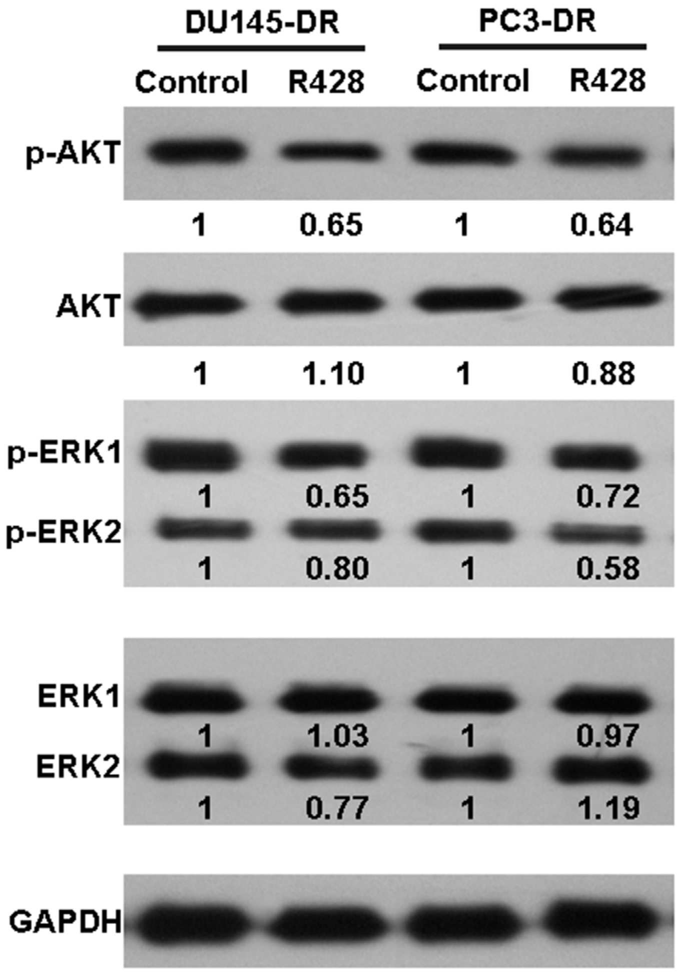

Supplementary Figure 6: PC3-DR and DU145-DR cells were treated with R428(1μM). The total and phosphorylation levels of ERK1/2 and AKT were analyzed using western blotting.
